# Cross-Correlation-Based Structural System Identification Using Unmanned Aerial Vehicles

**DOI:** 10.3390/s17092075

**Published:** 2017-09-11

**Authors:** Hyungchul Yoon, Vedhus Hoskere, Jong-Woong Park, Billie F. Spencer

**Affiliations:** 1 Department of Civil and Environmental Engineering, Michigan Technological University, Houghton, MI 49930, USA; hyung@mtu.edu; 2 Department of Civil and Environmental Engineering, University of Illinois at Urbana-Champaign, Urbana, IL 61801, USA; hoskere2@illinois.edu (V.H.); bfs@illinois.edu (B.F.S.); 3 School of Civil and Environmental Engineering, Urban Design and Studies, Chung-Ang University, Seoul 06974, Korea

**Keywords:** structural health monitoring, system identification, computer vision, Unmanned Aerial Vehicles

## Abstract

Computer vision techniques have been employed to characterize dynamic properties of structures, as well as to capture structural motion for system identification purposes. All of these methods leverage image-processing techniques using a stationary camera. This requirement makes finding an effective location for camera installation difficult, because civil infrastructure (i.e., bridges, buildings, etc.) are often difficult to access, being constructed over rivers, roads, or other obstacles. This paper seeks to use video from Unmanned Aerial Vehicles (UAVs) to address this problem. As opposed to the traditional way of using stationary cameras, the use of UAVs brings the issue of the camera itself moving; thus, the displacements of the structure obtained by processing UAV video are relative to the UAV camera. Some efforts have been reported to compensate for the camera motion, but they require certain assumptions that may be difficult to satisfy. This paper proposes a new method for structural system identification using the UAV video directly. Several challenges are addressed, including: (1) estimation of an appropriate scale factor; and (2) compensation for the rolling shutter effect. Experimental validation is carried out to validate the proposed approach. The experimental results demonstrate the efficacy and significant potential of the proposed approach.

## 1. Introduction

Structural system identification is the process of obtaining a model of a structural system based on a set of measurements of structural responses. Oftentimes, the responses of a structure such as displacements, accelerations, and strains are measured by traditional systems which usually require a tedious installation process or expensive equipment [1]. For example, linear variable differential transformers require fixed reference (e.g., scaffolds), and a GPS is either inaccurate or expensive (e.g., NDGPS) [2]. Accelerometers are reference-free, but inaccurate in reconstructing the DC component of the response [3].

Recently, computer vision-based techniques have been adopted to measure dynamic displacements for structural system identification purposes. Vision-based methods can measure multiple points on the structure, with a relatively simple installation and an inexpensive setup [3,4,5,6,7,8]. While the early stages of vision-based techniques focused on the measurement of the response itself, recent studies showed how these techniques could be used for system identification. Schumacher and Shariati [9] introduced the concept of a virtual visual sensor (VVS) that could be used for the modal analysis of a structure. Bartilson et al. [10] utilized a minimum quadratic difference (MQD) algorithm to analyze traffic signal structures. Feng and Feng [11,12] implemented upsampled cross correlation (UCC) and orientation code matching (OCM) for structural system identification using a vision sensor system. Chen et al. [13] used a motion magnification technique for modal identification. Yoon et al. [14] implemented a KLT tracker to identify a model for a laboratory-scale six-story building model using a smartphone and an action camera such as the GoPro camera. Feng and Feng [15] estimated stiffness reduction of a beam structure using vision-based displacements. Cha et al. [16] implemented damage detection of a cantilever beam using an unscented Kalman filter and vision-based, phase-based optical flow.

Despite the promising results of these vision-based approaches to utilizing cameras for structural system identification, all of these systems require cameras to be fixed to a stationary reference; this requirement limits their use for many civil infrastructure applications. For example, bridges are usually constructed over rivers, roads, or other obstacles, which makes finding an appropriate location for camera installation difficult. Also, the responses of high-rise buildings and some of their structural components can be difficult to capture with fixed camera.

Unmanned aerial vehicles (UAVs) or unmanned aircraft systems (UASs) may provide an opportunity to take a video of civil infrastructure more effectively by allowing the camera to get closer to the structure. Commercial UAV markets are growing dramatically, resulting in improved performance in terms of stability and mobility. Commercial-grade, off-the-shelf UAVs are now equipped with 4K resolution cameras. Capturing images of civil infrastructure from an aerial perspective using these UAVs offers the opportunity to resolve the issues with traditional fixed-reference, vision-based structural monitoring. Furthermore, UAVs can enable non-contact remote monitoring of a structure.

As opposed to the traditional way of using stationary cameras, the use of UAVs brings with it the issue of the motion of the camera. Because the camera is moving, the measured displacement will be relative to the UAV, not the absolute displacement. Yoon et al. [17,18] introduced an approach to estimate the motion of the camera and then to determine the absolute displacement of the structure; however, additional stationary targets needed to be identified in the scene. In addition, the scale factor for the measurement needed to be determined, as the UAV’s distance from the structure is not constant. Consequently, system identification results using the relative measurement will be erroneous. In addition, a rolling shutter effect of the CMOS sensors found in most commercial video cameras can be another source of error. Due to the sequential-readout structure of CMOS sensors, each scanline of the acquired image is exposed at a different time, resulting in geometric image distortion when the object or the video camera moves during image capture [19]. Because of the motion of UAVs, the effect of this rolling shutter effect will have increased significance compared to when the camera was stationary.

This paper proposes a new method for system identification using the relative displacements obtained directly from the UAV’s video images. In addition, the paper addresses two additional challenges: (1) changes in scale factor; and (2) rolling shutter effect. The scale factor is required to relate the pixel motions between frames to real world units. Because the scale factor is affected by the UAV’s motion, an adaptive scaling mechanism is introduced where the scale factor is recomputed for every frame. An additional compensation method is proposed to minimize the rolling shutter effect. The images are then processed to obtain accurate estimates of the displacement of the structure relative to the UAV. Subsequently, the cross-correlation functions are calculated for the various points on the structure, which effectively compensates for the motion of the UAV. Finally, these cross-correlations functions are used with the NExT-ERA for system identification. The proposed method is validated through an experiment on a six-story model building. The experiment compares three approaches for system identification which uses accelerometers, a stationary-camera, vision-based system, and the proposed vision-based system using an UAV. The comparison is made based on the accuracy of the extracted modal parameters in terms of natural frequencies and mode shapes.

## 2. Proposed Methodology

### 2.1. Overview

**Figure 1:**
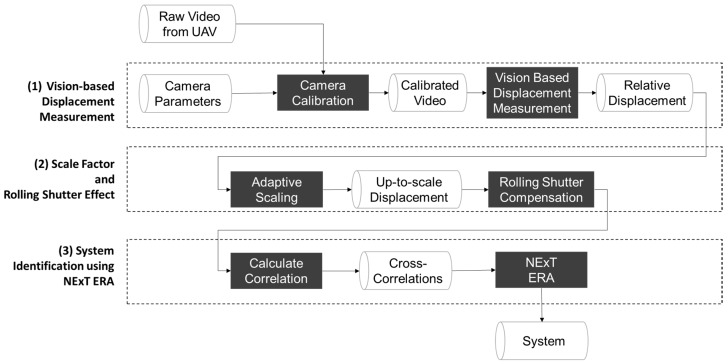
Overview of system identification using unmanned aerial vehicles (UAVs).

Figure 1 shows an overview of the proposed methodology. The underlying pipeline is composed of three main phases: (1) the conventional, vision-based method for displacement measurement proposed by Yoon et al. [14]; (2) novel methods for addressing the varying scale factor, rolling shutter effect, and; (3) the system identification method using relative displacements of a structure free from the UAV’s motion.

### 2.2. Conventional, Vision-Based Displacement Measurement

The first phase is to estimate the displacement of the structure relative to the UAV. This phase is based on the structural displacement measurement proposed by Yoon et al. [14]. The first step is camera calibration. Due to the imperfection of camera lenses, significant radial distortion exists in the raw video. The camera calibration module removes this distortion for achieving more accurate displacement measurement by applying the method proposed by Zhang [20]. Once the distortion is removed, the dynamic response of structures is determined by analyzing the calibrated video frame-by-frame. Once the region of the interests is selected by the user, feature points such as corner points are extracted by using the Harris corner detector [21]. Using the extracted feature points, relative displacements are calculated by applying the KLT tracker [22] while removing the outliers by MLESAC [23]. The result of the procedure will be a displacement of each story relative to the reference. This displacement vector ${\left[\begin{array}{cc} u_{i} & v_{i} \end{array}\right]}^{T}$—in pixel coordinates—will be converted into world coordinates and appropriately scaled as described in the following steps.

### 2.3. Displacement Measurement Using an UAV

**Figure 2:**
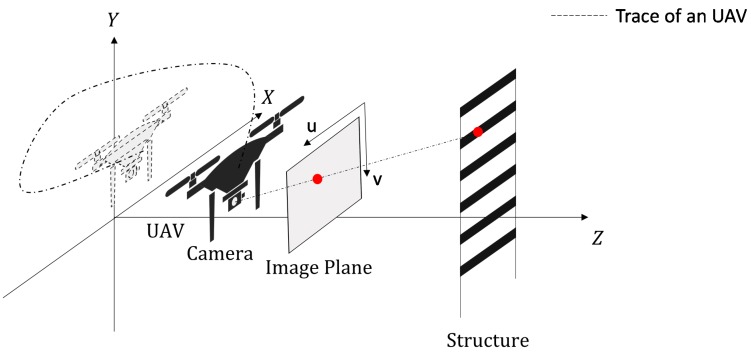
Motion of the UAV with respect to a target structure.

The 2D projected point [u, v] on an image plane obtained from a camera installed on an UAV for given 3D point in the world coordinate [X, Y, Z] (see Figure 2) can be expressed by using the pinhole camera model [24] as

(1) $$S\left[\begin{array}{c} u\\ v\\ 1 \end{array}\right]=\left[\begin{array}{ccc} \alpha_{x} & \gamma & u_{0}\\ 0 & \alpha_{y} & v_{0}\\ 0 & 0 & 1 \end{array}\right]\left[\begin{array}{cc} R_{3\times 3} & t_{1\times 3} \end{array}\right]\left[\begin{array}{c} X\\ Y\\ Z\\ 1 \end{array}\right]$$

where *λ* is an arbitrary value, R is a rotation matrix with 3 degree-of-freedom (i.e., $\theta_{x}$, $\theta_{y}$ and $\theta_{z}$), $t=[t_{x},\;t_{y}\text{​},\;t_{z}]$ is a UAV’s translation vector, *α_x_*, *α_y_* are the normalized focal length of a camera both directions, and *u*_0_ and *v*_0_ are the principle points. After applying the camera calibration, $\gamma ,\;u_{0},\;\;\mathrm{and}\;\;v_{0}$ can be set to zero, and $\alpha =\alpha_{x}=\alpha_{y};$ then, the projected 2D image point can be written as below:

(2) $$\begin{array}{ll} & \left[\begin{array}{l} u\\ v \end{array}\right]\\ =\frac{1}{S} & \left[\begin{array}{l} X(\cos\theta_{y}\cos\theta_{z})+Y(-\cos\theta_{x}\sin\theta_{z}+\sin\theta_{x}\sin\theta_{y}\cos\theta_{z})+Z(\sin\theta_{x}\sin\theta_{z}+\cos\theta_{x}\sin\theta_{y}\cos\theta_{z})+t_{x}\\ X(\cos\theta_{y}\sin\theta_{z})+Y(\cos\theta_{x}\cos\theta_{z}-\sin\theta_{x}\sin\theta_{y}\sin\theta_{z})+Z(-\cos\theta_{x}\sin\theta_{z}+\cos\theta_{x}\sin\theta_{y}\sin\theta_{z}+t_{y} \end{array}\right] \end{array}$$

where $S=\frac{-X\sin\;\theta_{y}+Y\sin\;\theta_{x}\cos\theta_{y}+Z\cos\theta_{x}\cos\theta_{y}+t_{z}}{\alpha }$ is a scale factor that will be derived in Section 2.4.

Assuming in-plane structural motion and no translation in the z-direction, the change in 2D relative displacement of a moving object with respect to the UAV’s motion at the *i*-th image frame can be written as follows

(3) $$\left[\begin{array}{c} d_{x,i}\\ d_{y,i} \end{array}\right]=S_{i+1}\left[\begin{array}{c} u_{i+1}\\ v_{i+1} \end{array}\right]-S_{i}\left[\begin{array}{c} u_{i}\\ v_{i} \end{array}\right]\;=\left[\begin{array}{c} \Delta X\left(\cos\theta_{y}\cos\theta_{z}\right)+\Delta Y(-\cos\theta_{x}\sin\theta_{z}+\sin\theta_{x}\sin\;\theta_{y}\cos\theta_{z})+t_{x}\\ \Delta X\left(\cos\theta_{y}\sin\theta_{z}\right)+\Delta Y(\cos\theta_{x}\cos\theta_{z}-\sin\theta_{x}\sin\;\theta_{y}\sin\theta_{z})+t_{y} \end{array}\right]$$

where ΔX and ΔY are the displacement of the structure between *i*-th and (*i* + 1)th image frame.

When the rotation angles are small, the Equation (3) can be re-written as below:

(4) $$\left[\begin{array}{c} d_{x,i}\\ d_{y,i} \end{array}\right]=S_{i+1}\left[\begin{array}{c} u_{i+1}\\ v_{i+1} \end{array}\right]-S_{i}\left[\begin{array}{c} u_{i}\\ v_{i} \end{array}\right]≅\left[\begin{array}{c} \Delta X+t_{x}\\ \Delta Y+t_{y} \end{array}\right]$$

As indicated by Equation (4), the change in relative displacement captured by the UAV is influenced by two factors: (1) scaling of the displacement related to S, and; (2) DC bias introduced by translational motion of the UAV (i.e., *t_x_* and *t_y_*). The scaling issue can be resolved by introducing an adaptive scale factor, as discussed in Section 2.4; the rolling shutter effect will be discussed in Section 2.5, and the influence of translational motion will be reduced using the Natural Excitation Technique (NExT) introduced in Section 2.6.

### 2.4. Adaptive Scale Factor

Most of the methods for measuring the displacement of the structure from a fixed camera on the ground assume the in-plane motion of the structure. This assumption allows for a constant scale factor for not only the entire frame but between frames as well. Given such a condition, the scale factor does not need to be determined at every frame to estimate the natural frequencies and mode shapes. However, this assumption is no longer valid for responses obtained through a moving camera, as in the case of the UAV, since there is bound to be movement in the Z-direction (i.e., $t_{z}$).

To address this issue, the scale factor for each frame can be calculated using the (1) known physical length of an object and (2) the pixel distance of the two corresponding points defined by the user. The distance of the two corresponding image points l can be expressed in terms of the known physical length of an object L by expanding the Equation (2).

(5) $$l=\|p_{1}-p_{2}\|=\frac{\alpha }{-X\sin\;\theta_{y}+Y\sin\;\theta_{x}\cos\;\theta_{y}+Z\cos\theta_{x}\cos\theta_{y}+t_{z}}L=\frac{1}{S}L$$

where $p_{1}$ and $p_{2}$ are the two end points of the object with a known physical length. ${\left\|\cdot \right\|}_{2}$ is the L2-norm of a vector. Note that α and Z are constant, and the angles are small, so the scale factor *S* will be a function of camera motion in the Z-direction.

The scale factor *S* in the initial image frame can be determined by dividing the length of the known object L by the pixel distance of the object in image frame *l*_0_. Based on the initial scale factor *S*_0_, the scale factor in each frame can be calculated using two designated feature points in the image as:

(6) $$S_{0}=\frac{L}{l_{0}}\;S_{i}=\frac{S_{0}\|p_{0,1}-p_{0,2}\|}{\left\|p_{i,1}\;-p_{i,2}\right\|}$$

where $p_{0,1}$ and $p_{0,2}$ are the two points having the maximum distance among the feature points in the initial frame and $p_{i,1}$ and $p_{i,2}$ are the two corresponding feature points in frame i.

### 2.5. Rolling Shutter Compensation

Besides adjusting for a scale factor in each frame, the rolling shutter effect that causes geometric distortions such as skew should be compensated. The rolling shutter (RS) is one type of image data acquisition that scans each row of the image sequentially using the complementary metal-oxide semiconductor (CMOS) sensors [19]. In the RS, due to the nature of sequential readout, if the relative velocity of a UAV and the object is large, the error become dramatic; compensation should be made to enable accurate system identification.

**Figure 3:**
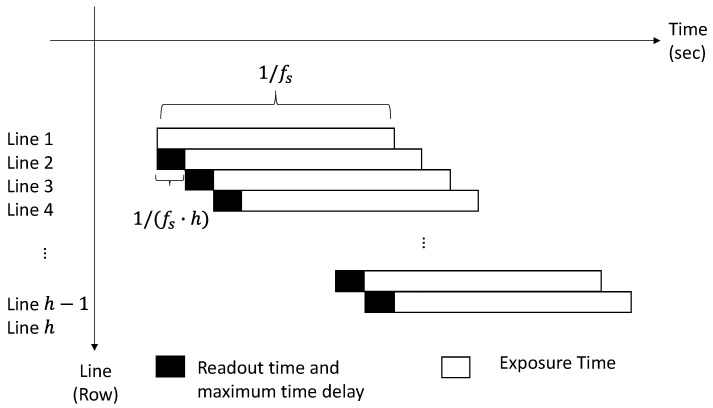
Readout time and exposure time in rolling shutter in single image frame.

Figure 3 illustrates the readout time and exposure time of each scanning line (row) in the image in the CMOS sensor. The readout time is the time required to digitize a single array of pixels and is determined by the speed of the A/D conversion and the number of rows in an image frame. The exposure time is the length of time when the CMOS sensor is exposed to light. Assuming the frame-rate $f_{s}$ of the camera is constant, the readout time and the maximum processing time delay can be expressed as

(7) $$t_{r}=\frac{1}{f_{s}h}$$

where $t_{r}$ is the readout time and maximum time delay for each line, and h is the height (total number of lines) of the image.

The distortion, in terms of the number of pixels, and due to the rolling shutter in the *i*-th line compared to the first line, $d_{i,RS},$ can then be written as the following equation:

(8) $$d_{i,RS}=t_{r}(i-1)V_{rel}$$

where $V_{rel}$ is the relative velocity in the direction where the rolling shutter occurs between the camera and the structure. $V_{rel}$ was obtained by the optical-flow using each sequential image frame. To compensate for the effect of the RS, the $d_{i,RS}$ was subtracted from the feature points at the *i*-th line.

### 2.6. Natural Excitation Technique (NExT) for System Identification

As described in the Section 2.2, the displacement obtained by a UAV denoted in Equation (4) is comprised of both the movement of the target structure and of the UAV. Rather than identifying the UAV’s movement to get absolute structural displacement, the proposed method seeks to use relative displacements from multiple locations of interest on the structure to conduct system identification.

To conduct system identification using the multiple relative displacements of the target structure obtained by a UAV, the cross-correlations between the relative displacements are used for system identification. Provided that the natural frequencies of the target structure do not overlap with the frequencies induced by the UAV’s non-stationary drift, the correlation between the relative displacements can be used for structural system identification.

Consider two 2-D displacements D*_i_* extracted at a point designated *i* in the image as:

(9) $$D_{i}=\left[\begin{array}{cc} x_{i}(t)+t_{x}(t)+m_{i}(t) & 0\\ 0 & y_{i}(t)+t_{y}(t)+n_{i}(t) \end{array}\right]$$

where D*_i_* is the decoupled matrix of 2D displacement; $x_{i}(t)$, $y_{i}(t)$ are the true absolute displacements of a target structure in the x and y axis at a point *i* in the image; $t_{x}(t)$, $t_{y}(t)$ are the translational movements of a UAV; and $m_{i}(t)$ and $n_{i}(t)$ are measurement noise in the x and y axis, respectively. The cross-correlation functions between $D_{1}$, $D_{2}$ are evaluated as

(10) $$\begin{array}{l} R_{D_{1}D_{2}}=\\ E[\left[\begin{array}{cc} x_{1}(t+\tau )+t_{x}(t+\tau )+m_{1}(t+\tau ) & 0\\ 0 & y_{1}(t+\tau )+t_{y}(t+\tau )+n_{1}(t+\tau ) \end{array}\right]\left[\begin{array}{cc} x_{2}(t)+t_{x}(t)+m_{2}(t) & 0\\ 0 & y_{2}(t)+t_{y}(t)+n_{2}(t) \end{array}\right]]\\ =\left[\begin{array}{cc} R_{x_{1}x_{2}}+R_{x_{1}t_{x}}+R_{x2t_{x}}+R_{t_{x}t_{x}} & 0\\ 0 & R_{y_{1}y_{2}}+R_{y_{1}t_{y}}+R_{y2t_{y}}+R_{t_{y}t_{y}} \end{array}\right] \end{array}$$

Provided that the dynamic translational movement of a UAV does not have correlation with the relative displacements of a structure, the cross-correlation $R_{D_{1}D_{2}}$ between the two 2D relative displacements can be expressed as

(11) $$R_{D_{1}D_{2}}\;=\left[\begin{array}{cc} R_{x_{1}x_{2}}+R_{t_{x}t_{x}} & 0\\ 0 & R_{y_{1}y_{2}}+R_{t_{y}t_{y}} \end{array}\right]$$

Because the cross-correlation between the translational motion (i.e., $R_{t_{x}t_{x}}$ or $R_{t_{y}t_{y}}$) does not involve the dynamic characteristics of the target structure, the cross-correlation between relative displacements can be used for system identification using a natural excitation technique (NExT), output-only modal analysis (James, et al. [25]). The NExT uses the correlation function of $R_{D_{i}D_{ref}}(\tau )$ to express the structural equation of motion in terms of M, C, and K as:

(12) $$M{\ddot{R}}_{D_{i}D_{\mathrm{ref}}}(\tau )+C{\dot{R}}_{D_{i}D_{\mathrm{ref}}}(\tau )+KR_{D_{i}D_{\mathrm{ref}}}(\tau )=0$$

where, $R_{D_{i}D_{\mathrm{ref}}}(\tau )$ is the correlation function between the relative displacement obtained at point *i* and at the reference point in the image , and M, C, and K are the mass, damping, and stiffness matrices, respectively.

**Figure 4:**
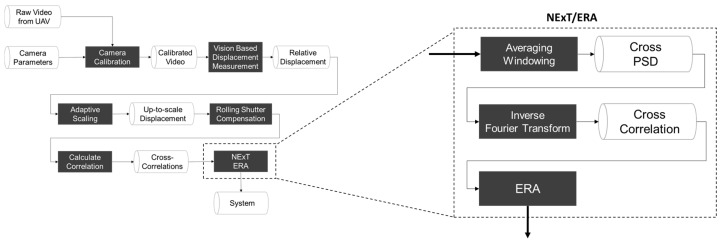
Flowchart for natural excitation technique (NExT)/eigensystem realization algorithm (ERA).

To extract the structural modal properties, the eigensystem realization algorithm (ERA) was applied after the NExT (see Figure 4), in which the cross-correlations between multiple relative displacements were obtained by applying the inverse Fourier transform of the cross power spectral density (CPSD) functions.

## 3. Experimental Validation

### 3.1. Experimental Setup

**Figure 5:**
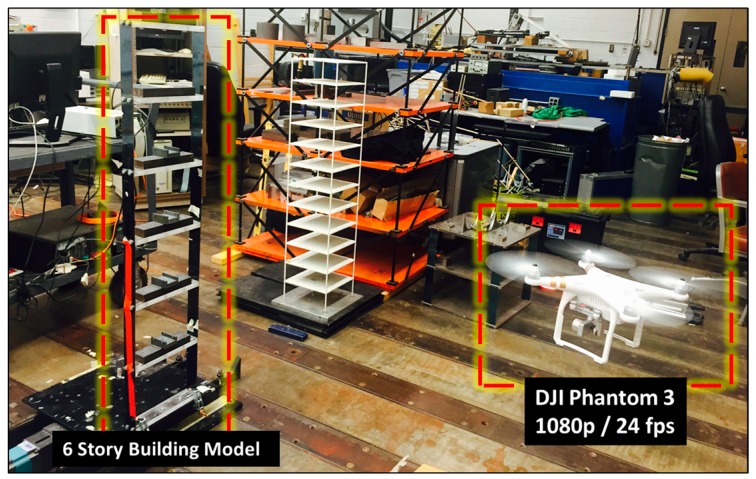
Experimental setup.

An experiment was designed to verify the proposed method. More specifically, the purpose was to verify whether the proposed method could allow a commercial grade UAV and an attached camera to accurately conduct system identification of a target structure by effectively reducing the error caused by the varying scale factor and the rolling shutter effect. A lab-scale test was carried out on a six-story building model. The six-story building model is a shear building that has equal masses at every floor and the building model is fixed on a uni-directional shaking table (see Figure 5). The six natural frequencies of the shear building model are 1.657, 5.04, 8.14, 10.83, 12.93, and 14.34 Hz. To excite the various structural vibration modes, a band-limited white noise (BLWN) was used as input motion during this experiment.

The DJI Phantom 3, a commercial grade UAV was used for the test. The camera installed on the UAV has 1080 p resolution and a frame rate of 25 fps. Also, the gimbal that holds the camera to the UAV embeds an accelerometer and a gyroscope to stabilize the motion of the camera against 3-axis rotation. The UAV was hovering by maintaining a 2 m distance from the structure to avoid collision.

As a reference, a stationary camera, an LG smartphone G3, was installed on the ground 1 m away from the structure to extract the absolute structural displacements. The video in the stationary camera was originally recorded at 60 fps with 1080 p and resampled at 25 fps for comparison. In addition, accelerometers were deployed on each story of the shear building model to compare the system identification results. The sampling rate for the acceleration was 1024 Hz.

### 3.2. Analyzed Adaptive Scale Factor

**Figure 6:**
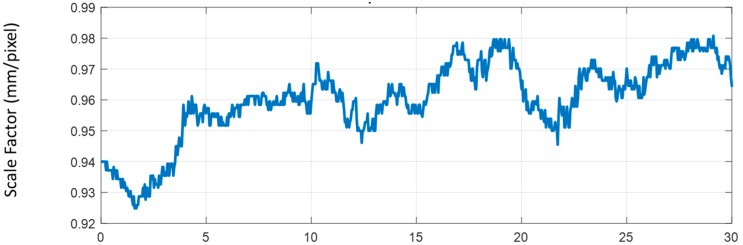
Change in the scale factor over time.

As discussed in the Section 2.3, the scale factor for the each image taken by the camera changes over time, due to the z-direction motion of the UAV. The scale factor was calculated for each frame automatically using the proposed method. Figure 6 shows that the scale factor varied from 0.925 to 0.981 during 30 s of the video, leading to maximum of 5.7% error in displacement conversion from pixel to mm.

### 3.3. Comparison of the Dynamic Responses

**Figure 7:**
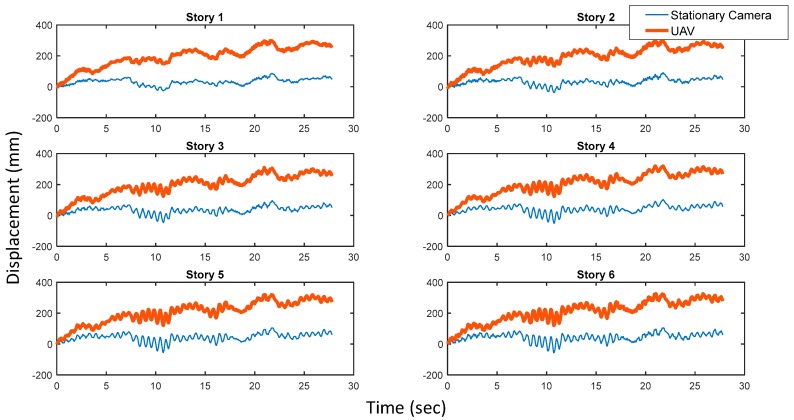
Comparison of absolute displacements and relative displacements.

Using the proposed procedure, the relative displacements of the six-story building model using a UAV were obtained, analyzed, and compared with the ones from a stationary camera on the ground. Figure 7 shows that there was large discrepancy between the relative displacements obtained from the UAV and the absolute displacements obtained from a stationary camera. It is easy to conclude that the motion of the UAV causes the drifts occurring in all six stories, since the trends are consistent across the floors.

**Figure 8:**
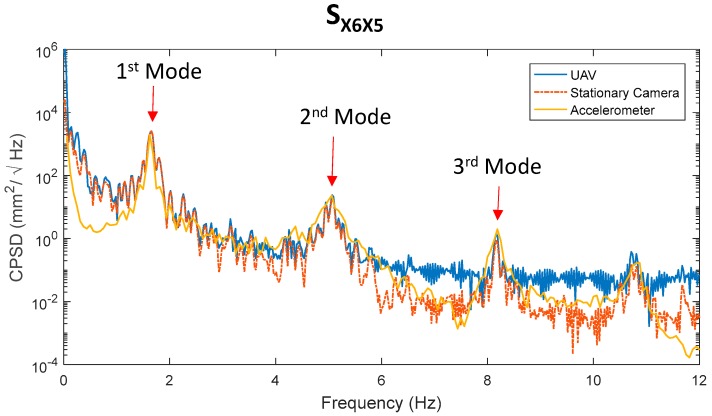
Comparison of cross power spectral density (CPSD) from the unmanned aerial vehicles (UAV) and references.

Figure 8 shows the CPSD of responses from the 5th floor and 6th floor of the shear building model from the UAV and two reference measurements from the stationary camera and accelerometers. Figure 8 shows that the cross power spectral densities from the UAV agreed very well with the reference in the frequency domain by showing three clear natural frequencies, while the relative displacements obtained from the UAV were significantly different compared to the absolute displacements from the stationary camera. Note that the CPSD obtained from the UAV had a large discrepancy in the DC component, with references due to the motion of the UAV.

**Figure 9:**
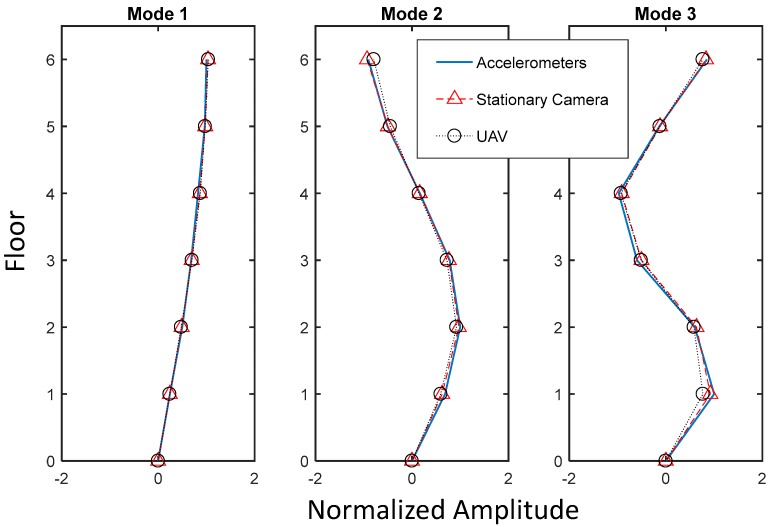
Comparison of mode shapes.

Based on the CPSD, NExT-ERA was then performed to conduct system identification; Table 1 and Figure 9 compare the results, in which the following observation can be made.

- Assuming that the system identification result from the accelerometers was the most reliable, the result from both the stationary camera and the UAV showed around 1% of maximum error in terms of natural frequency estimation. The comparison indicates that the proposed method using the UAV provides as accurate natural modes as those obtained from the stationary camera and accelerometers.
- Mode shapes extracted by the proposed method using the UAV were compared with ones from the stationary camera and the accelerometers in terms of the mode assurance criteria (MAC) value; all three mode shapes showed over 99% consistency when compared with the reference, and are shown in Figure 9.

## 4. Discussion

The proposed method was able to cancel out the three translational motion (i.e., $t_{x}$, $t_{y}$ and $t_{z}$) without directly calculating the camera motion. As shown in Section 2, the adaptive scale factor introduced in Equation (6) could resolve the issue of out-of-plane motion (Z-direction) of the camera. The cross-correlations of the projected displacement introduced in Equation (11) could cancel out the other two translation motions (X and Y direction) of the camera.

In this study, the validation test was conducted at an indoor laboratory where wind affect was negligible. Due to the environment, the assumption of the small rotational motion of the camera was able to hold. However, when subjected to strong wind, the assumption of small rotational motion cannot hold anymore, and thus the correlation will not be invariant to the camera motion. For example, displacements at each story will be subjected to the same amount of translation when the rotational motion is negligible. On the other hand, the displacement of each story will be subjected to a different amount of translation when the rotational motion is large, resulting in inconsistent correlations. Therefore, in such cases, a method calculating absolute displacement such as Yoon et al. [14] should be applied, even though the method requires additional stationary points and a longer calculation time.

## 5. Conclusions

This paper presents a vision-based method for system identification using consumer-grade cameras in a UAV to conduct system identification. Unlike traditional, vision-based system identification, three major problems arise due to the non-stationary motion of the UAV. These are: (1) change in scale factor; (2) the rolling shutter effect; and (3) conducting system identification using the relative displacement obtained from the aerial camera. To address these issues, novel image-processing techniques for compensating errors and a cross-correlation-based system identification process were proposed in the paper.

An image-processing technique is applied to extract relative structural displacements when the camera is subjected to the nonstationary motion of a UAV. Based on the information extracted from vision-based displacement measurements, the signal is corrected in two steps. First, adaptive scaling is developed and applied to update the time-varying scale factor with each frame. Second, a rolling shutter compensation is applied which compensates for the image distortion due to progressive scanning of CMOS sensors. However, the displacements extracted from the camera in the UAV still contain not only the motion of the target structure but also the motion induced by the movement of the UAV. The proposed method eliminates the effect of motion of the UAV for system identification by employing the NExT-ERA method, which uses cross-correlation between the extracted displacements.

An experimental test was carried out on a six-story shear building model with the UAV to validate the proposed method. A stationary camera and accelerometers were employed to obtain reference measurement. The shear building model was excited by a shaking table with band-limited white noise. The experimental results show that the change in scale factor due to the out-of-plane motion of the UAV (up to 5.7%) was successfully compensated through the proposed method. Also, the results of system identification indicate that the proposed UAV-based method estimated the natural frequency, with a maximum error of 1%, and the mode shapes, with MAC value of as low as 99.67, for mode shape extraction when compared with the reference obtained from the accelerometers. The experimental results show that the proposed method has the potential to identify the natural frequencies and the mode shapes with reasonable levels of accuracy.

The proposed method can estimate the modal properties of a target structure without identifying the location of a UAV in real-time for system identification, which is highly expensive in computation. However, careful attention must be paid to the hovering motion of the UAV, which accounted for 0–1 Hz in the frequency range. As the UAV’s motion is predominantly below 0.5 Hz, it is likely to miss the natural frequencies that are located below 0.5 Hz.

## Figures and Tables

**Table 1:** Comparison of system identification results.

Natural Frequencies (Hz)				MAC (%)		Error (%)	
Mode	Accelerometers (Reference)	Stationary Camera	UAV	Stationary Camera	UAV	Stationary Camera	UAV
1	1.632	1.649	1.649	99.99	99.99	1.04	1.04
2	5.054	5.060	5.043	99.99	99.86	0.12	0.22
3	8.175	8.166	8.170	99.99	99.67	0.11	0.06
